# SARS-CoV-2 nsp1: Bioinformatics, Potential Structural and Functional Features, and Implications for Drug/Vaccine Designs

**DOI:** 10.3389/fmicb.2020.587317

**Published:** 2020-09-29

**Authors:** Yuan-Qin Min, Qiong Mo, Jun Wang, Fei Deng, Hualin Wang, Yun-Jia Ning

**Affiliations:** ^1^State Key Laboratory of Virology, Wuhan Institute of Virology, Center for Biosafety Mega-Science, Chinese Academy of Sciences, Wuhan, China; ^2^University of Chinese Academy of Sciences, Beijing, China

**Keywords:** SARS-CoV-2, COVID-19, coronavirus, nsp1, pathogenic factor, bioinformatics, structure and function analysis, drug and vaccine development

## Abstract

The emerging coronavirus disease (COVID-19) caused by SARS-CoV-2 has led to social and economic disruption globally. It is urgently needed to understand the structure and function of the viral proteins for understanding of the viral infection and pathogenesis and development of prophylaxis and treatment strategies. Coronavirus non-structural protein 1 (nsp1) is a notable virulence factor with versatile roles in virus-host interactions and exhibits unique characteristics on sequence, structure, and function mode. However, the roles and characteristics of SARS-CoV-2 nsp1 are currently unclear. Here, we analyze the nsp1 of SARS-CoV-2 from the following perspectives: (1) bioinformatics analysis reveals that the novel nsp1 is conserved among SARS-CoV-2 strains and shares significant sequence identity with SARS-CoV nsp1; (2) structure modeling shows a 3D α/β-fold of SARS-CoV-2 nsp1 highly similar to that of the SARS-CoV homolog; (3) by detailed, functional review of nsp1 proteins from other coronaviruses (especially SARS-CoV) and comparison of the protein sequence and structure, we further analyzed the potential roles of SARS-CoV-2 nsp1 in manipulating host mRNA translation, antiviral innate immunity and inflammation response and thus likely promoting viral infection and pathogenesis, which are merited to be tested in the future. Finally, we discussed how understanding of the novel nsp1 may provide valuable insights into the designs of drugs and vaccines against the unprecedented coronavirus pandemic.

## Introduction

Severe acute respiratory syndrome coronavirus 2 (SARS-CoV-2; previously named 2019 novel coronavirus, 2019-nCoV) is the causative agent of the coronavirus disease 2019 (COVID-19), an emerging, highly infectious pneumonia that has rapidly spread worldwide and caused severe social and economic disruption (World Health Organization [WHO], 2020; Wu et al., 2020; Zhou et al., 2020; Zhu et al., 2020). Up to July 16th 2020, more than 13.81 million confirmed cases have been reported around the world according to the COVID-19 Dashboard by the Center for Systems Science and Engineering at Johns Hopkins University (Dong et al., 2020).

 Coronaviruses (CoVs) belong to the *Coronaviridae* family of the *Nidovirales* order and are grouped into four genera, namely, α-CoV, β-CoV, γ-CoV, and δ-CoV (International Committee on Taxonomy of Viruses). The former two genera are mainly found in mammals while the latter ones are predominantly detected in birds (Woo et al., 2012; Cui et al., 2019). SARS-CoV-2 is a novel β-CoV and likely originated from bats (Ceraolo and Giorgi, 2020; Zhou et al., 2020). CoVs are single-stranded, positive sense RNA viruses with the genome size ranging from 26 to 32 kb (Woo et al., 2010). The 5′-proximal two-third of the genome contains the first large open reading frames (ORFs 1a and 1b) encoding precursor polyproteins (pp1a and pp1ab), which are processed into 15 or 16 non-structural proteins (nsps) by the ORF1a-encoded viral proteases (Ziebuhr, 2005; Perlman and Netland, 2009). These nsps are thought to play pivotal and multiple roles in virus replication (Ziebuhr, 2005; Perlman and Netland, 2009; Enjuanes et al., 2016; de Wilde et al., 2018). Therein, nsp1 is the most N-terminal cleavage product, which notably possesses the following special properties. Firstly, nsp1 only exits in α-CoVs and β-CoVs, while γ-CoVs and δ-CoVs do not encode nsp1, indicating that nsp1 is likely to be involved in the evolution of CoVs (Snijder et al., 2003; Connor and Roper, 2007; Woo et al., 2010). Secondly, nsp1 sequences are highly divergent for different species even in the same genus, whereas several structural studies revealed that the nsp1 proteins from α-CoVs and β-CoVs likely share similar β-barrel folds along with α-helices (Almeida et al., 2007; Jansson, 2013; Shen et al., 2018). Furthermore, nsp1 has no homologs among cellular or viral proteins other than in CoVs (Connor and Roper, 2007). Noticeably, series of studies showed that nsp1 is likely a critical virulence factor involved in CoV infection and pathogenesis and hence a promising target for antiviral research (Wathelet et al., 2007; Zust et al., 2007; Jimenez-Guardeno et al., 2015; Narayanan et al., 2015). These unique features together with the important functions attract our interest to characterize the novel nsp1 encoded by the emerging SARS-CoV-2 from its sequence to the potential structure and function. Also, we discussed the possible insights brought by the understanding of the nsp1 into antiviral drug and vaccine development to combat the unprecedented coronavirus pandemic.

## Bioinformatics of SARS-CoV-2 nsp1

**FIGURE 1:**
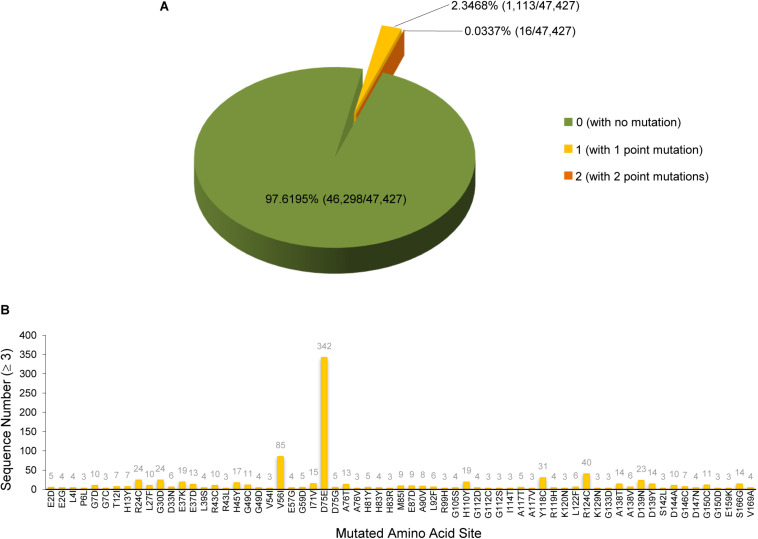
SARS-CoV-2 nsp1 sequence analysis. SARS-CoV-2 genomic sequences of high quality (47,427 in total) were collected from the GISAID database. Sequence alignment was performed using the EMBOSS diffseq program with the strain Wuhan-Hu-1 as the reference. According to the alignment, the nsp1 region was found to be highly conservative. Most of the nsp1 sequences are identical to that of the Wuhan-Hu-1 strain without any mutation. Only a few nsp1 protein sequences (1,129 of the analyzed 47,427 sequences) have one or two point mutations, compared to the reference strain. The detailed numbers and ratios of the nsp1 protein sequences with or without mutation were displayed in panel **(A)**. The identified mutation sites and the numbers (≥3) of the sequences harboring the indicated point mutation were summarized in panel **(B)**. Note that most of the mutations were found in only one or two sequences and are not shown in panel **(B)**. See also Supplementary Table S1 and the descriptions in the text.

**FIGURE 2:**
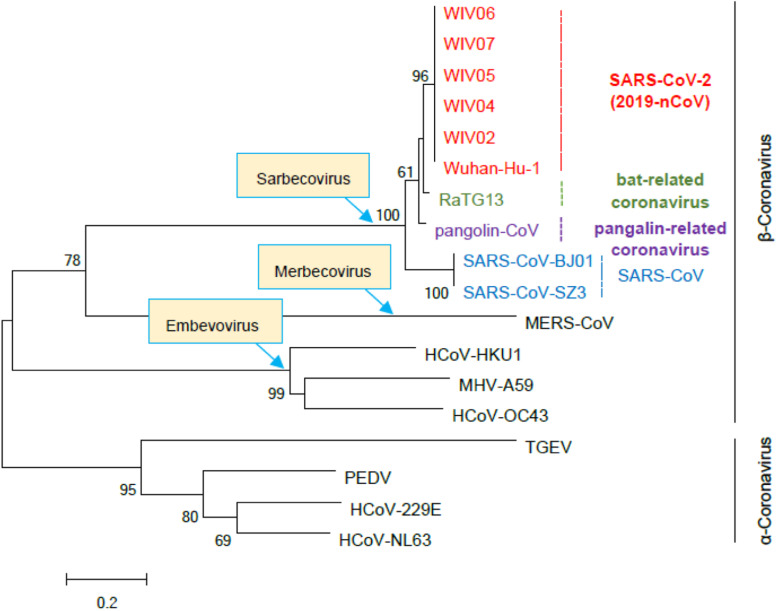
Phylogenetic tree based on the amino acid sequences of β- and α-CoV nsp1. The nsp1 sequences of the indicated representative β- and α-CoVs were collected and aligned with MEGA 6.0. The phylogenetic tree was constructed by the Neighbor-Joining algorithm with MEGA 6.0 and tested with 1000 bootstrap replicates. Numbers on the tree indicate percentage bootstrap values (>50%) for the associated nodes. The scale bar represents the number of substitutions per site. Accession numbers of the sequences used for analysis are as follows: SARS-CoV-2 strains Wuhan-Hu-1 (EPI_ISL_402125), WIV02 (EPI_ISL_402127), WIV04 (EPI_ISL_402124), WIV5 (EPI_ISL_402128), WIV6 (EPI_ISL_402129), and WIV7 (EPI_ISL_402130); SARS-CoV strains BJ01 (AY278488) and SZ3 (AY304486); bat-related coronavirus RaTG13 (EPI_ISL_402131); pangolin-related coronavirus (pangolin-CoV, EPI_ISL_410721); Middle East respiratory syndrome coronavirus (MERS-CoV; NC019843); Human coronavirus HKU1 (NC006577); Human coronavirus OC43 (AY391777); Mouse hepatitis virus strain A59 (MHV-A59, NC_001846.1); Transmissible gastroenteritis virus (TGEV, ABD58989.1); Porcine epidemic diarrhea virus (PEDV, AJP67455.1); Human coronavirus 229E (NC002645); Human coronavirus NL63 (NC_005831).

**FIGURE 3:**
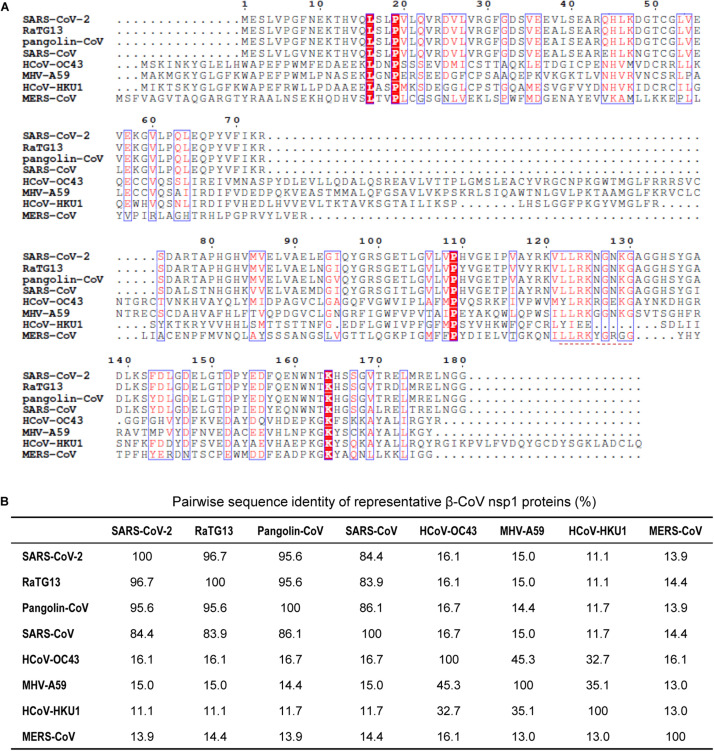
Comparison of representative β-CoV nsp1 sequences. **(A)** Amino acid sequence alignment of nsp1 encoded by several representative β-CoVs were performed by Clustal X Version 2.1 and viewed by ESPript. The numbers above the aligned sequences indicate the amino acid sites of SARS-CoV-2 nsp1. The amino acids conserved in all the analyzed nsp1 sequences are shown in white with red background. The residues conserved in most of the analyzed sequences are shown in red and boxed in blue. A region highly conserved among β-CoVs (corresponding to amino acids 122-130 of SARS-CoV-2 nsp1) is indicated with a red dashed line. **(B)** Summary of the sequence identity percentage of β-CoV nsp1 in pairwise comparison. Virus strains of SARS-CoV-2 and SARS-CoV used here are Wuhan-Hu-1 and SZ3, respectively.

Based on the study of the polyprotein cleavage in other β-CoVs (Snijder et al., 2003; Harcourt et al., 2004; Prentice et al., 2004; Ziebuhr, 2005), the papain-like proteinase residing in nsp3 should process the replicase polyprotein of SARS-CoV-2 at glycine-180/alanine-181 (G180/A181) cleavage site, resulting in the production of SARS-CoV-2 nsp1 containing 180 amino acids (aa) in length with a calculated molecular weight of ∼19.8 kDa. The detailed physicochemical parameters for SARS-CoV-2 nsp1 analyzed by the ExPASy ProtParam tool (Gasteiger et al., 2005) are listed in Table 1 in comparison with those for SARS-CoV nsp1. SARS-CoV-2 genomic sequences of high quality submitted to the GISAID databank (as of June 18, 2020) were next collected and subjected to analysis of sequence alignment. The alignment showed that among the analyzed 47,427 sequences, 46,298 (>97.6%) are identical in the aa sequence of nsp1 without any mutation (Figure 1A and Supplementary Table S1). Even for the potential mutants identified, no one contains point mutations on more than two sites and actually, only 16 nsp1 sequences harbor changes on two aa sites (Figure 1A and Supplementary Table S1). Additionally, most of the possible mutations (if not sequencing error) only were found in 1 or 2 sequences and even the most frequently detected D75E mutation was seen in only 342 sequences (∼0.7%) (Figure 1B and Supplementary Table S1), indicating very low frequency of mutation. These analyses suggest that the SARS-CoV-2 strains are highly conserved in the nsp1 region, reflecting the necessity of this non-structural protein for SARS-CoV-2 biology. To evaluate the relationship of SARS-CoV-2 nsp1 with the homologs from other CoVs, a phylogenetic tree based on the aa sequence was estimated. Consistent with the evolutionary analysis of viral genomes (Wu et al., 2020; Zhang et al., 2020; Zhou et al., 2020; Zhu et al., 2020), SARS-CoV-2 nsp1 were clustered with the homologs from bat-related RaTG-13, pangolin-related CoV, and SARS-CoV in the *Sarbecovirus* group (Figure 2). Further, as demonstrated by the sequence alignment analysis, SARS-CoV-2 nsp1 shares 84.4, 96.7, and 95.6% sequence identities with the nsp1 proteins of SARS-CoV, RaTG13, and pangolin-CoV, respectively, but has quite low identities with those encoded by the β-CoVs in other subgenera (Figures 3A,B).

**TABLE 1:** Physicochemical properties of SARS-CoV-2 and SARS-CoV nsp1.

Parameter^a^	SARS-CoV-2 nsp1	SARS-CoV nsp1
Number of aa	180	180
Molecular weight Theoretical pI	19775.31 5.36	19640.12 5.35
Formula	C_872_H_1383_N_247_O_270_S_4_	C_8__6__2_H_138__2_N_24__6_O_27__2_S_3_
No. of negatively charged residues	27 (Asp + Glu)	26 (Asp + Glu)
No. of positively charged residues	19 (Arg + Lys)	18 (Arg + Lys)
Estimated half-life^b^	30 h	30 h
Instability index	28.83 (classified as stable)	27.31 (classified as stable)
Aliphatic index	89.72	97.89
Grand average of hydropathicity	−0.378	−0.357

*^a^The physicochemical parameter values of nsp1 were deduced by the ProtParam tool using the strains SARS-CoV-2 Wuhan-hu-1 and SARS-CoV BJ01 as the references, respectively. ^b^Predicted in the context of mammalian reticulocyte model in vitro.*

## Structural Modeling of SARS-CoV-2 nsp1

**FIGURE 4:**
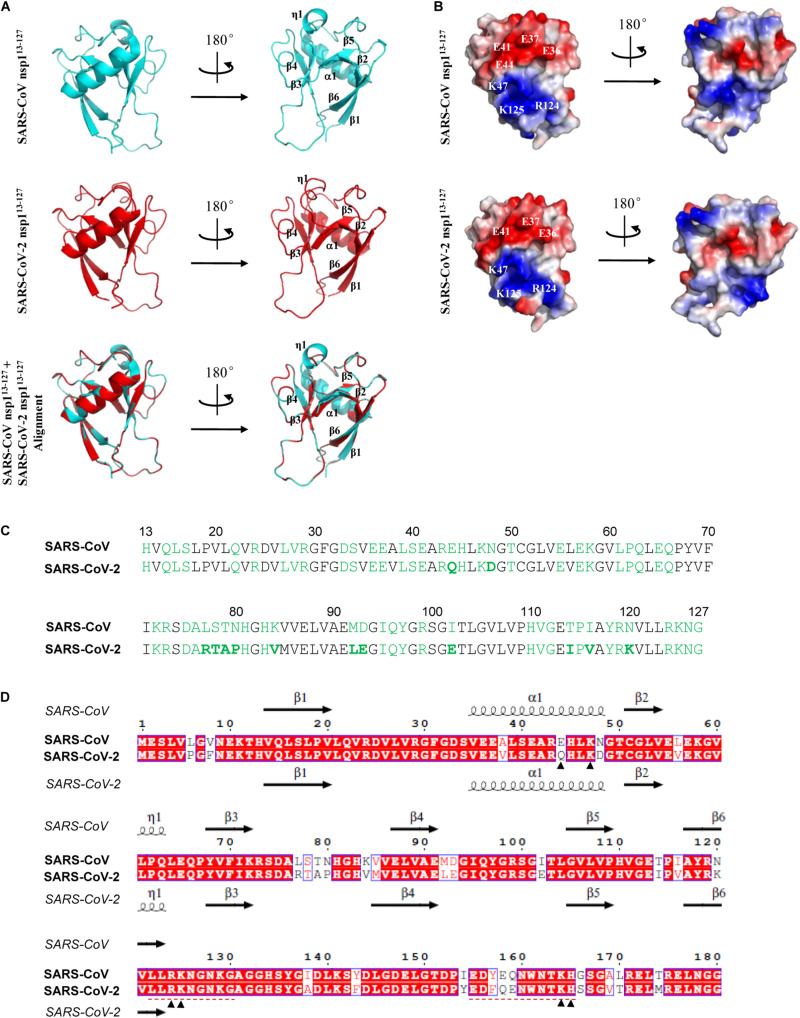
Predicted structure features of SARS-CoV-2 nsp1. **(A)** Ribbon models of the SARS-CoV and SARS-CoV-2 nsp1 structures. The determined NMR structure of SARS-CoV nsp1^13– 127^ (residues 13–127) was downloaded from Protein Data Bank (PDB code: 2HSX) as shown in cycan. The structural modeling of SARS-CoV-2 nsp1^13– 127^ was conducted by target-template alignment using SWISS-MODEL server with the structure of SARS-CoV nsp1^13– 127^ as template. Visualization of structural details and alignment of the two structures (cyan overlapping red) were performed by PyMOL software. The RMSD value between the predicted SARS-CoV-2 nsp1^13– 127^ and SARS-CoV nsp1^13– 127^ structures is 0.087 Å. The organization of the main secondary structures is indicated in a selected rotation. **(B)** Solid surface views of the SARS-CoV nsp1^13– 127^ and predicted SARS-CoV-2 nsp1^13– 127^ structures. These models have the same orientations as in panel **(A)**. Red represents negatively charged; blue represents positively charged. Some key surface residues in the large areas of negative or positive charges are indicated. **(C)** Solvent-exposed residues of SARS-CoV and SARS-CoV-2 nsp1 proteins are highlighted in green. Those in boldface represent a few differences between SARS-CoV and SARS-CoV-2 nsp1. **(D)** Comparison of aa sequences and secondary elements between SARS-CoV and SARS-CoV-2 nsp1. Sequence alignment was conducted using Clustal X and visualized by ESPript. The main secondary structures of SARS-CoV and SARS-CoV-2 nsp1 are displayed above and below the sequences, respectively. The secondary structures of SARS-CoV-2 were predicted by SWISS-MODEL server as described in panel **(A)**. Note that some predicted secondary structures exhibit subtle differences with those analyzed in PyMOL. Several aa sites discussed in the text were indicated with arrowheads. Two regions involved in the studies of attenuated vaccines were underlined with red dashed lines.

At present, the crystal structures of nsp1 from two α-CoVs, porcine epidemic diarrhea virus (PEDV) and transmissible gastroenteritis virus (TGEV), and a nuclear magnetic resonance (NMR) structure of the major segment of SARS-CoV nsp1 (residue 13-127) have been determined (Almeida et al., 2007; Jansson, 2013; Shen et al., 2018). Although the sequence identities of PEDV nsp1 with those of TEGV and SARS-CoV are only 23 and 12%, respectively, these nsp1 proteins intriguingly share similar core structures, with the root mean square deviation (RMSD) values of 1.8 Å (PEDV nsp1 vs. TEGV nsp1) and 2.4 Å (PEDV nsp1 vs. SARS-CoV nsp1) (Shen et al., 2018). Thus, CoV nsp1 proteins likely fold into similar core structures despite the low sequence homology. Given the significant sequence identity of the nsp1 between SARS-CoV-2 and SARS-CoV, a predicted structure for the major region of the novel nsp1 of SARS-CoV-2 based on the information of the SARS-CoV homolog would be highly credible. To conduct the comparative modeling analysis, the amino acid sequence of SARS-CoV-2 nsp1 was firstly subjected to searching in the Protein Data Bank (PDB). As expected, the NMR structure of SARS-CoV nsp1^13–127^ (PDB: 2HSX) was identified as the template for 3D structure construction of SARS-CoV-2 nsp1 on the SWISS-MODEL server (Schwede et al., 2003). As shown in the results of structural modeling, SARS-CoV-2 nsp1^13–127^ exhibits similar α/β-folds with SARS-CoV nsp1^13–127^ that consist of a characteristic six-stranded β-barrel and a long α1-helix covering one opening of the barrel (Figure 4A). As with the sequence and structure similarity, the solvent-exposed residues which would be available to mediate contacts with other molecules are largely conserved (Figures 4B,C). Notably, an uneven electrostatic charge distribution also can be observed on the SARS-CoV-2 nsp1 protein surface (Figure 4B). These large areas of positive or negative charges (Figure 4B) could mediate intermolecular interactions by electrostatic forces, which may contribute to the protein function. Particularly, the K47, R124, and K125 conservative on the surface of the two nsp1 form a positive tunnel (Figures 4B–D) that could potentially mediate RNA binding and be involved in the inhibition of host mRNA translation by the viral protein (as further discussed below). The RMSD value of the predicted SARS-CoV-2 nsp1^13–127^ with SARS-CoV nsp1^13–127^ is 0.087 Å, indicating few differences between the two structures (Figure 4A). Some subtle differences on a few surface residues (such as the E44 of SARS-CoV nsp1 versus Q44 of SARS-CoV-2 nsp1) lead to slight discrepancy of the detailed surface electrostatics (Figures 4B–D), which however, does not disrupt the notable feature of the uneven surface charge distribution (Figure 4B). Overall, the core structure of SARS-CoV-2 nsp1 could be very similar to that of SARS-CoV nsp1, providing further support for the likely conservative protein functions. Additionally, two independent studies very recently reported a short C-terminal structure of SARS-CoV-2 nsp1 (aa 153–179) containing two helices that may play an important role in host protein synthesis inhibition (Schubert et al., 2020; Thoms et al., 2020) (see further discussion below). However, the N-terminal major region of the viral protein failed to be solved in these analyses based on cryo-electron microscopy. Combination of the finding of the C-terminal helix domain with the insights of the larger N-terminal core structure predicted here may help inform better understanding of the entire structure of nsp1.

## Potential Function of SARS-CoV-2 nsp1

Severe acute respiratory syndrome coronavirus nsp1 is a most well characterized nsp1 of CoVs in terms of its biological functions and action mode. Previous studies have shown that SARS-CoV nsp1 is likely a critical virulent factor with multiple effects on the virus-host interaction interface, such as inhibiting host mRNA translation (Kamitani et al., 2006; Wathelet et al., 2007; Zust et al., 2007; Tohya et al., 2009; Huang et al., 2011a; Lokugamage et al., 2015; Narayanan et al., 2015), antagonizing IFN signaling (Wathelet et al., 2007; Zust et al., 2007), and inducing inflammatory cytokines and chemokines (Law et al., 2007; Pfefferle et al., 2011). Moreover, it should be noted that some of these effects of nsp1 are likely conserved in other β-CoVs and even α-CoVs, despite the low protein sequence homology. Given the high sequence identity and the very likely structural similarity between SARS-CoV-2 nsp1 and SARS-CoV nsp1, the knowledge of SARS-CoV nsp1 functions could be highly instructive for understanding the biological roles of SARS-CoV-2 nsp1. Here, we analyzed the potential functions of SARS-CoV-2 nsp1 by systematically referring to the observed nsp1 activities of SARS-CoV (together with the conservativeness of the corresponding functions among other β- or α-CoVs) and by detailedly comparing the critical aa sites or motifs involved in the particular functions.

### Inhibition of Host mRNA Translation

It has been reported that SARS-CoV nsp1 binds to the 40S ribosomal subunit and inhibits host mRNA translation using a two-pronged strategy (Kamitani et al., 2009). On the one hand, by interacting with the 40S ribosomal subunit, SARS-CoV nsp1 suppresses the translation of capped cellular mRNAs through the blockade of the steps involved in 48S and 80S initiation complex formation (Kamitani et al., 2009; Lokugamage et al., 2012). On the other hand, the nsp1 induces an endonucleolytic RNA cleavage in the 5′-UTR of host mRNAs perhaps by recruiting a cellular endonuclease, subsequently resulting in accelerated degradation of the cleaved mRNA intermediate by the cellular Xrn1-mediated mRNA decay pathway (Kamitani et al., 2009; Huang et al., 2011b; Gaglia et al., 2012). Interestingly, although SARS-CoV nsp1 may also inhibit the viral mRNA translation to some extent by the association with 40S ribosomal subunit, it does not induce SARS-CoV mRNA cleavage (Huang et al., 2011b; Tanaka et al., 2012). Mechanistically, the presence of the stem-loop 1 (SL1) in the 5′-UTR which can bind to nsp1 may protect the SARS-CoV mRNAs from nsp1-induced endonucleolytic RNA cleavage, leading to partial circumvention of the nsp1-mediated translational inhibition and hence ensuring sufficient viral protein expression (Huang et al., 2011b; Tanaka et al., 2012). Notably, inhibition of host mRNA translation seems to be a common function of nsp1 from both representative β- and α-CoVs (Wang et al., 2010; Narayanan et al., 2015), although the underlying mechanisms are likely various in line with the low sequence identity. For example, TGEV nsp1 also efficiently suppresses protein synthesis in mammalian cells, whereas it does not bind to the 40S ribosomal subunit or promote host mRNA degradation (Huang et al., 2011a). Middle East respiratory syndrome coronavirus (MERS-CoV) nsp1 can negatively regulate host gene expression by inhibiting host mRNA translation and inducing cleavage and degradation of host mRNAs as well (Lokugamage et al., 2015). However, MERS-CoV nsp1 does not bind to the 40S ribosomal subunit either (Lokugamage et al., 2015). Moreover, different from the cytoplasmic localization of SARS-CoV nsp1, MERS nsp1 is distributed in both nucleus and cytoplasm and selectively targets host mRNAs transcribed in the nucleus for host protein synthesis inhibition but spares mRNAs of cytoplasmic origin (Lokugamage et al., 2015). Given the significant sequence and structural similarities of the nsp1 proteins from SARS-CoV-2 and SARS-CoV and the high conservativeness of the function among CoVs, SARS-CoV-2 nsp1 highly likely has a capacity to inhibit host mRNA translation similar to SARS-CoV nsp1. Moreover, the aa sites of SARS-CoV nsp1, K164 and H165, critical for both inhibition of translation initiation complexes and cleavage of host mRNA (Lokugamage et al., 2012) are conservative in SARS-CoV-2 nsp1 (Figure 4D). Further, the aa sites R124 and K125 required specifically for the host mRNA cleavage but not for the targeting of ribosomal complexes (Lokugamage et al., 2012) also are conserved in SARS-CoV-2 nsp1 and similarly participate in the formation of the positively charged area on the SARS-CoV-2 nsp1 surface that potentially could bind to host mRNA targets (Figures 4B–D). These features support that the function and mechanism for translational inhibition are likely shared by the two nsp1 proteins. Indeed, as aforementioned, a translational inhibition by SARS-CoV-2 nsp1 has been demonstrated in two very recent studies (Schubert et al., 2020; Thoms et al., 2020) during the submission of the present paper. A short C-terminal domain that contains two helices respectively consisting of residues 154–160 and 166–179 appeared to be inserted into the entrance region of the ribosomal mRNA channel, likely contributing to the translational blockade (Schubert et al., 2020; Thoms et al., 2020). However, many important questions therein still remain open. For instance, whether SARS-CoV-2 nsp1 induces endonucleolytic mRNA cleavage, whether the viral mRNA translation can escape from the nsp1 activity, and how the major globular region of nsp1 at the N-terminus exactly contributes to the translation inhibition are all unknown. As specific shut-off of host protein synthesis that can block the normal physiological processes of host cells including antiviral responses and lead to increased viral replication and cytopathy could be a major aspect of the viral pathogenesis, this potential function of SARS-CoV-2 nsp1 and the underlying mechanisms are still merited to be systematically investigated in the future study.

### Antagonism of Host Antiviral Innate Immunity

Similar to SARS-CoV, SARS-CoV-2 seems to induce only low levels of antiviral IFNs (types I and III IFNs) in cell and animal models and in patients (Acharya et al., 2020; Blanco-Melo et al., 2020), reflecting effective viral counteraction of host innate immune responses. SARS-CoV nsp1 has been shown to be an important antagonist of host antiviral responses which not only mediates the general repression of host protein synthesis including those of IFNs (as mentioned above) but also seems to interfere with virus- and IFN-triggered antiviral signaling cascades specifically (Kamitani et al., 2006; Wathelet et al., 2007; Narayananj et al., 2008; Jauregui et al., 2013). Antiviral responses of IFN system consist of two aspects, IFN induction triggered by virus infection and IFN signaling initiated by binding of secreted IFNs to their cell surface receptors. Wathelet et al. (2007) reported that SARS-CoV nsp1 could inhibit virus-infection-stimulated IFN expression (i.e., IFN induction) by attenuating the signaling pathways meditated by transcription factors ATF2/c-Jun, NF-κB, and IRF3/IRF7 although the direct host target(s) interacting with nsp1 is(are) unknown. The authors further showed that the nsp1 could also suppress IFN signaling by specifically decreasing STAT1 phosphorylation while exhibiting little effect on the activation of STAT2 or the related Janus kinases (Wathelet et al., 2007). Interestingly, the aa sites R124 and K125 are also critical for the inhibitory activities of SARS-CoV nsp1 against IFN induction and signaling (Wathelet et al., 2007). A mutant virus strain harboring an engineered nsp1 with R124S/K125E mutations induced stronger antiviral responses and was substantially attenuated in the ability of replication, compared to the wild type virus (Wathelet et al., 2007). Further, two C-terminal regions of nsp1 were later found to be important to inhibit IFN responses as well (Jimenez-Guardeno et al., 2015): one is aa 122–130 (LLRKNGNKG) that is identical between SARS-CoV and SARS-CoV-2 and even highly conserved among β-CoVs (Figures 3A, 4D), and the other is aa 155–165 that contains the conserved K164 and H165 (Figure 4B). Additionally, Jauregui et al. (2013) generated and analyzed a series of SARS-CoV nsp1 mutants, targeting solvent exposed residues, and identified several classes of mutants that differentially altered nsp1 inhibition of host protein expression and/or antiviral signaling. These studies could further help inform the detailed elucidation of the SARS-CoV-2 nsp1 function. Likewise, the repression of antiviral IFN responses also seems to be a common function of the nsp1 encoded by pathogenic or potentially pathogenic α- and β-CoVs (Narayanan et al., 2015). As antagonizing host antiviral responses and particularly the IFN system responses should be a key activity of nsp1 as the viral virulence factor, it warrants a prioritized and systematic investigation in the future work as well.

### Potential Roles in Inflammatory Dysregulation

Exuberant proinflammatory cytokine and chemokine production accompanied by impaired antiviral innate immune responses likely is the defining feature of COVID-19 and drives the disease development (Acharya et al., 2020; Blanco-Melo et al., 2020; Soy et al., 2020). Similarly, SARS-CoV infection could also lead to the immune dysregulation characterized with excessive stimulation of various inflammatory cytokines along with hampered antiviral IFN responses (Channappanavar et al., 2016; Kindler and Thiel, 2016; Wong et al., 2016). Coincidently, SARS-CoV nsp1 has been shown to stimulate chemokine production in human lung epithelial cells (Law et al., 2007) while inhibiting antiviral IFN induction and signaling, indicating that nsp1 might be involved in the immune and inflammatory dysregulation during SARS-CoV infection. Of note, the induction of chemokines by nsp1 expression appeared to be dependent on the NF-κB signaling (Law et al., 2007), although the viral protein has been shown to interfere with NF-κB activation in the IFN induction pathway triggered by viral infection (Wathelet et al., 2007). Thus, the detailed mechanisms underlying these nsp1 actions remain to be further investigated. In addition, by employing a genome-wide yeast two-hybrid screening, Pfefferle et al. (2011) identified immunophilins (cyclophilins and FK506-binding proteins) as interaction partners of SARS-CoV nsp1. These immunophilins are known to modulate the Calcineurin/NFAT pathway that plays an important role in immune cell activation. Consistently, the authors then showed that nsp1 expression (by transfection) and live SARS-CoV infection both strongly increased the Calcineurin/NFAT signaling and hence the induction of interleukin-2, further supporting the possible involvement of nsp1 in the virus-induced inflammatory dysregulation (Pfefferle et al., 2011). More intriguingly, the Calcineurin/NFAT pathway might be a common requirement for efficient replication of not only the beta-CoVs (different SARS-CoV strains) but also other α- and even γ-CoVs, as the inhibitor of the Calcineurin/NFAT signaling, Cyclosporin A (CspA), robustly suppressed the replication of all the tested CoVs (Pfefferle et al., 2011). Therefore, it will be interesting and significant to examine the interaction of SARS-CoV-2 nsp1 with immunophilins and the role of the Calcineurin/NFAT pathway in SARS-CoV-2 infection, for not only better understanding of nsp1 function and COVID-19 pathogenesis but also the development of antiviral therapeutics targeting the virus-host interaction (see below for further discussion).

### Other Possible Activities of nsp1

**FIGURE 5:**
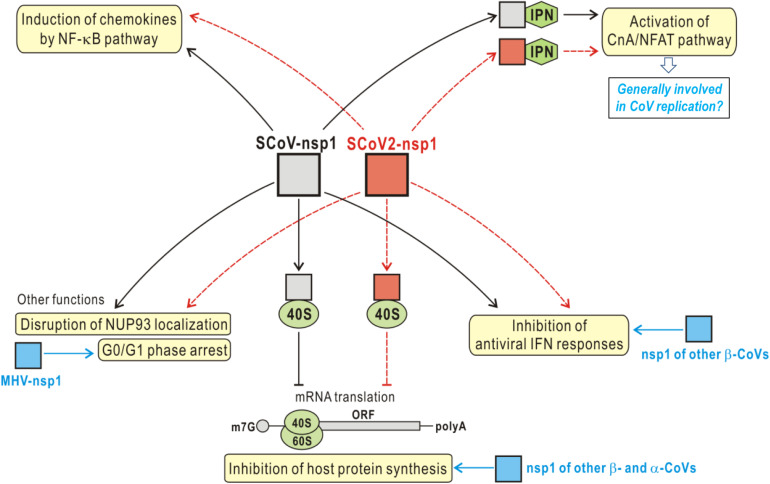
Potential functions of nsp1. The reported functions of SARS-CoV nsp1 (SCoV-nsp1) include inhibition of host protein synthesis by binding to 40S ribosomal subunit, inhibition of antiviral IFN responses, activation of CnA/NFAT signaling by interacting with immunophilins (IPN), induction of chemokines, disruption of NUP93 localization, and induction of cell cycle arrest in G0/G1 phase (summarized in the yellow textboxes). Notably, some of these functions are shared by the nsp1 of other representative CoVs (indicated in blue), despite the low sequence identifies of the nsp1 proteins. In addition, the cellular CnA/NFAT pathway also seems to be generally involved in CoV replication, as inhibition of the pathway could block the replication of not only SARS-CoV (β-CoV), but also the CoVs from other genera. Given the high sequence identity and the possible structural similarity, SARS-CoV-2 nsp1 (SCoV2-nsp1) likely has the activities similar to those of SCoV-nsp1, which merit systematic investigations in the future for better understanding of the viral infection and pathogenesis and development of drugs or vaccines. See also the descriptions in the text.

In addition to the major functions discussed above, nsp1 may have other biological activities including modulation of cell cycle and biomolecular nuclear-cytoplasmic transport, as suggested in the previous studies of SARS-CoV and other CoVs (Chen et al., 2004; Wathelet et al., 2007; Narayanan et al., 2015; Gomez et al., 2019). Wathelet et al. (2007) reported that the expression of SARS-CoV nsp1 inhibited cell proliferation and increased the ratio of the cells in G0/G1 phase without affecting cell viability, although the underlying mechanism is unknown. Interestingly, the nsp1 of MHV was shown to induce G0/G1 cell cycle arrest as well (Chen et al., 2004), indicating one more potentially common role of CoV nsp1. Additionally, Gomez et al. (2019) recently showed that SARS-CoV nsp1 appeared to disrupt the localization of NUP93, a member of the nuclear pore complex, on the nuclear envelope and thus specifically affect the nuclear-cytoplasmic distribution of a cellular protein nucleolin. Moreover, the conservative R124 and K125 seemed to be important for nsp1 targeting of Nup93 and nucleolin, although the further biological significance for this action was not investigated yet (Gomez et al., 2019). These observations demonstrate the versatile roles of nsp1 as a remarkable pathogenic factor. The potential functions of nsp1 that could be validated first in the future study are summarized in Figure 5.

## Implications for Drug/Vaccine Designs

Given the role of nsp1 as the possibly major pathogenic factor, this viral protein in turn could be a promising target for drug design, as inhibition of nsp1 or its functions might directly reduce the pathogenic effects of the viral protein (e.g., the host cell shutoff and inflammatory induction effects) or indirectly attenuate viral pathogenicity by increasing host antiviral responses or viral susceptibility to the antiviral responses. With nsp1 as a target, drugs could be screened and designed to act on either the nsp1 molecule itself or the host factors and biological processes involved in nsp1-cell interactions. Targeting the latter could potentially reduce the possibility of the emergence of mutational resistance and lead to the development of broad-spectrum treatment strategies. As aforementioned, the study on the host factors interacting with SARS-CoV nsp1 by Pfefferle et al. (2011) revealed the possibly general dependence of not only SARS-CoV but also other pathogenic CoVs on the cellular cyclophilins and the Calcineurin/NFAT pathway. Correspondingly, the peptide inhibitor CspA that binds to cyclophilins to suppress the Calcineurin/NFAT signaling and is used clinically to prevent organ rejection of transplant patients potently blocked the replication of all the tested α-, β-, and γ-CoVs at low micromolar concentrations (Pfefferle et al., 2011). Thus, drugs like CspA and particularly those without immunosuppressive activity (Fischer et al., 2010) could be recommended for tests of anti-SARS-CoV-2 efficacy. Very recently, Gordon et al. (2020) investigated the host cell interactome of SARS-CoV-2 proteins and thereby identified series of pharmacological agents with efficient antiviral activity in cell infection model, presenting a good example for drug discovery based on virus-host protein-protein interactions. However, the expression level of nsp1 appeared to be very low in the study (Gordon et al., 2020), which should be a result of the inhibitory effect of nsp1 on protein expression including that of its own in the transient transfection assays (Kamitani et al., 2006; Narayananj et al., 2008; Lokugamage et al., 2012). Correspondingly, the identification of nsp1-interacting proteins seemed to be insufficient and no druggable target against nsp1-cell interactions were found in the study (Gordon et al., 2020). Further exploration of the viral protein-host interactions with improved approaches for interactome identification may reveal more targets for development of host-directed therapeutic strategies against the SARS-CoV-2 infection.

Also, the knowledge of the nsp1 functions might help inform vaccine development. For instance, recombinant viruses with mutated nsp1 incapable of counteracting host antiviral responses could be used for design of attenuated strains. Live-attenuated vaccines are considered more effective compared to other types of vaccines, as they could produce high levels of antigenic stimulation and induce long-term, balanced immune responses (Lambert et al., 2005; Graham et al., 2013). As mentioned above, Wathelet et al. demonstrated that a recombinant SARS-CoV with a mutated nsp1 (R124S/K125E) was largely attenuated in cultured cells while inducing stronger antiviral responses (Wathelet et al., 2007). Based on a deletion in the nsp1-coding sequence along with reverse genetics, another study by Zust et al. (2007) presented an example for design of an attenuated coronavirus vaccine. The authors reported that the recombinant coronavirus MHV mutant encoding the truncated nsp1 (with deletion of 829–927 nt) was greatly attenuated *in vivo*, demonstrating the role of nsp1 as the major pathogenicity factor (Zust et al., 2007). Importantly, even low doses of the nsp1 mutant MHV elicited effective cellular immune responses and protected mice against homologous and heterologous virus infections (Zust et al., 2007). Similarly, Lin et al. (2013) later reported that MHV could also be highly attenuated *in vivo* by deletion of the conserved LLRKxGxKG region of nsp1 (Figure 3A) and moreover, the recombinant virus with this mutation completely protected mice from lethal virus challenge. In addition to single gene modification, nsp1 engineering could be used in combination with the directed mutation of other viral genes for design of much safer and more stable live-attenuated vaccines. Regarding this, the study by Jimenez-Guardeno et al. (2015) provided a notable example. A SARS-CoV mutant with the E gene deleted was attenuated in animal models and provided full protection against virulent virus challenge; however, the mutant virus regained fitness by genomic insertion mutations after serial passages in cultured cells or *in vivo*, exhibiting instability (Jimenez-Guardeno et al., 2015). Jimenez-Guardeno et al. (2015) next showed that mutant SARS-CoV strains with either of the two C-terminal regions of nsp1 (aa 122–130 or 155–165) deleted were attenuated, accompanied by stronger induction of IFN responses (as aforementioned), and importantly these viruses induced successful protection against lethal viral challenge, indicating these nsp1 mutant viruses are potential vaccine candidates. Further, recombinant SARS-CoV mutants with deletions in both nsp1 and E genes were generated and demonstrated to be fully attenuated and genetically stable during passages in both cell and animal models (Jimenez-Guardeno et al., 2015). Moreover, vaccination with these attenuated mutant strains including two safety guards completely protected mice against lethal viral challenge (Jimenez-Guardeno et al., 2015). The study might present a paradigm of attenuation strategy for obtaining highly efficient, safe and stable coronavirus vaccine candidates (Jimenez-Guardeno et al., 2015). Together, these data and analyses may provide valuable information for the development of attenuated vaccines against SARS-CoV-2.

## Conclusion

As a potentially significant pathogenic factor, nsp1 merits more attentions in the future studies on viral protein functions and action modes. Functional and mechanistic elucidation of SARS-CoV-2 nsp1 would not only promote the understanding of the COVID-19 pathogenesis but also help inform the development of drugs and vaccines against this emerging infectious disease. Although the nsp1 proteins between α- and β-CoVs have no evident sequence homology, they likely share a highly similar core 3D structure characterized with unique β-barrel folds, in accordance to several determined nsp1 structures. Thus, given the high sequence identity of the nsp1 proteins encoded by SARS-CoV-2 and SARS-CoV, the predicted α/β-fold structure of SARS-CoV-2 nsp1 that exhibits few differences with the reported structure of SARS-CoV nsp1 could be highly instructive. Likewise, the main functions known for SARS-CoV nsp1 including inducing host cell shut-off and inhibiting antiviral IFN responses are also conserved in many other pathogenic CoVs (Figure 5), despite the variances of the underlying mechanisms. Therefore, the functions of SARS-CoV nsp1 discussed in this article (especially the activities to inhibit host protein synthesis and antiviral IFN responses and to enhance Calcineurin/NFAT signaling) could be shared by SARS-CoV-2 nsp1, which warrants the future systematic tests in priority. On the other hand, in spite of the significant sequence identity of the nsp1 proteins from SARS-CoV-2 and SARS-CoV, they still have some differences in ∼28 aa sites. It will be also interesting to investigate whether and to what extent these differences could affect the structure, function, and action mode of the viral proteins. Further, would they somehow be related to the possibly different infectivity and pathogenicity of the two viruses? The distinct epidemiological features of COVID-19 in comparison with the previous SARS-CoV epidemic likely reflect some differences of SARS-CoV-2 and SARS-CoV in infection and pathogenicity which could be attributed to various viral and host factors and their complex interactions. Comparative studies of the important viral proteins like the two nsp1 encoded by SARS-CoV-2 and SARS-CoV, together with systematic analyses of both viral and cellular factors, would benefit better understanding of the potential differences in viral infectivity and pathogenicity. According to the alignment analysis of the collected SARS-CoV-2 genomic sequences, nsp1 is highly conserved among the viral strains. Nonetheless, variances of SARS-CoV-2 nsp1 sequences still should be closely watched as they might one day further influence the viral infectivity and pathogenicity.

## Data Availability Statement

All datasets presented in this study are included in the article/Supplementary Material.

## Author Contributions

HW, FD, and Y-JN supervised the research. Y-JN conceived the work. Y-QM, QM, Y-JN, and JW performed the bioinformatics and modeling analyses. Y-QM, QM, and Y-JN created the figures and assessed the data. Y-JN, Y-QM, and QM reviewed and abstracted the data from the literatures. Y-QM and Y-JN wrote the draft with input from other authors. Y-JN reviewed and revised the manuscript. All authors reviewed the results, read and approved the final manuscript version.

## Conflict of Interest

The authors declare that the research was conducted in the absence of any commercial or financial relationships that could be construed as a potential conflict of interest.
